# Pigeons as Carriers of Clinically Relevant Multidrug-Resistant Pathogens—A Clinical Case Report and Literature Review

**DOI:** 10.3389/fvets.2021.664226

**Published:** 2021-05-24

**Authors:** Dorota Chrobak-Chmiel, Ewelina Kwiecień, Anna Golke, Beata Dolka, Krzysztof Adamczyk, Małgorzata J. Biegańska, Marina Spinu, Marian Binek, Magdalena Rzewuska

**Affiliations:** ^1^Department of Preclinical Sciences, Institute of Veterinary Medicine, Warsaw University of Life Sciences, Warsaw, Poland; ^2^Department of Pathology and Veterinary Diagnostics, Institute of Veterinary Medicine, Warsaw University of Life Sciences, Warsaw, Poland; ^3^Department of Infectious Diseases and Preventive Medicine, Law and Ethics, University of Agricultural Sciences and Veterinary Medicine, Cluj-Napoca, Romania

**Keywords:** antimicrobial resistance, *Candida albicans*, *Escherichia coli*, MRSA, pigeon

## Abstract

Pigeons are widespread bird species in urban regions (*Columba livia* forma *urbana*) and may carry pathogens with zoonotic potential. In recent years, more and more data indicate that these zoonotic pathogens are multidrug resistant. Our results confirmed that global trend. Three different multidrug-resistant pathogens were isolated from an oral cavity of a racing pigeon with lesions typical for pigeon pox virus infection. *Staphylococcus aureus* was recognized as methicillin resistant, thus resistant to all beta-lactams. Additionally, it was also resistant to many other classes of antibiotics, namely: aminoglycosides, tetracyclines, phenicols, lincosamides, and macrolides. *Escherichia coli* showed resistance to all antimicrobials tested, and it was classified as intermediate to amikacin. Moreover, *Candida albicans* resistant to clotrimazole, natamycin, flucytosine, and amphotericin and intermediate to ketoconazole, nystatin, and econazole was also isolated. This raises the question how pigeons acquire such highly resistant strains. Therefore, more data are needed concerning the resistance to antibiotics in strains from domestic and wild pigeons in Poland. Until the problem is fully understood, it will be challenging to implement adequate planning of any control measures and check their effectiveness.

## Introduction

In pigeons, most staphylococcal infections are caused by *Staphylococcus aureus*; however, a few studies have indicated that after *S. aureus*, the most prevalent coagulase-positive staphylococci (CoPS) in pigeons are *Staphylococcus delphini* and *Staphylococcus intermedius* (1, 2), which inhabit the choanal slit (posterior nasal apertures) of healthy birds.

*S. aureus* is widely spread among humans and numerous animal species. It means that it can be easily transmitted between animals and humans. Since pigeons share common environment with humans, they may not only be the source of staphylococcal infection but may also pose a reservoir of bacteria-carrying resistance and virulence factor genes. Therefore, this might be of a great importance in the context of public health.

Extensive and often inappropriate use of antimicrobials causes a strong selective pressure that leads to the rapid increase in antimicrobial resistance in bacteria. Thus, the antibiotic use plays a crucial role in the emerging public health crisis of antimicrobial resistance. Increased number of multidrug-resistant bacteria has become a global problem. The World Health Organization (WHO) alarms that humanity is at risk of returning to the “pre-antibiotic era” (3). It should be noted that resistant bacteria may circulate among humans, animals, and the environment. Therefore, the “One World—One Health” concept created in 2004 becomes an especially important issue nowadays (4, 5).

Homing pigeons and fancy pigeons, which are bred for ornamental traits are very popular in Poland. Currently, homing pigeons are mainly used in racing competitions. Nowadays, there is a huge problem in Poland related to the frequent use of antimicrobials by breeders without consulting a veterinarian (6, 7). This directly contributes to the increase of drug resistance in bacteria occurring in pigeons.

## Methods

**Figure 1:**
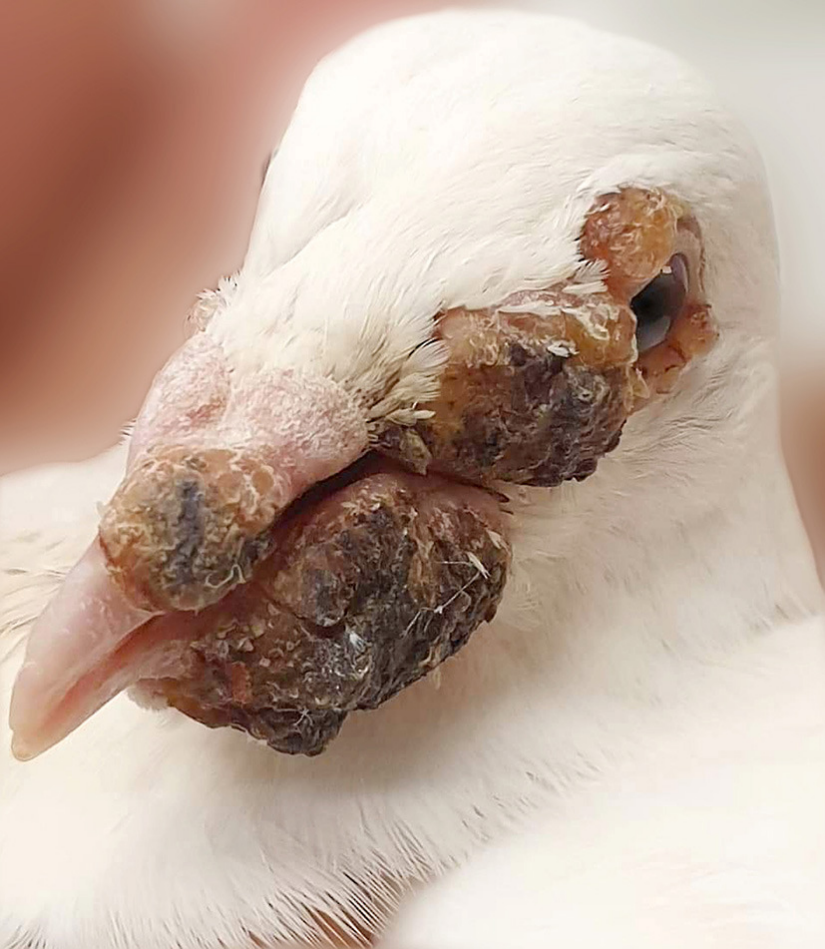
Pox in pigeon from which multidrug-resistant *E. coli* and *S. aureus* strains were isolated: note typical yellow-to-brown nodules on and around beak and eyes.

In August 2019, one racing pigeon from the affected pigeon loft was submitted to the veterinary clinic. Clinical examination revealed several dry, yellowish nodular lesions on the eyelids, as well as protuberant black pocks in the nostrils, cere region, and lower beak. Lesions were firmly attached to the skin. In addition, the abscess was found on the palate. Clinical examination allowed the recognition of pigeon pox virus infection based on the presence of typical cutaneous and mucosal diphtheritic lesions (Figure 1). The swab from oral cavity was collected for laboratory tests. Basing on the clinical changes, bacteriological as well as mycological examinations were performed. Collected swab was cultured on Columbia agar supplemented with 5% sheep blood (Graso Biotech, Poland), MacConkey agar (Graso Biotech, Poland), and Sabouraud agar (Biomerieux, France). Bacterial isolates were identified based on their phenotypic properties, such as: Gram stain characteristics, catalase and oxidase results, as well as on colony morphology on blood agar and MacConkey agar plates. For further identification of staphylococcal isolate, a tube coagulase test was performed. Additionally, a rapid agglutination test was used for the differentiation of *S. aureus* by the detection of clumping factor and protein A specific for this staphylococcal species (Microgen Staph, Graso Biotech, Poland). Moreover, for tested staphylococcal strain multiplex PCR assay based on the amplification of *nuc* gene was used. This method allows for differentiation of coagulase-positive staphylococci isolated from animals (8). Four reference strains from the Culture Collections of the University Göteborg *S. intermedius* CCUG 6520^T^, *S. schleiferi* subsp. *coagulans* CCUG 37248^T^, *S. delphini* CCUG 30107^T^, and *S. pseudintermedius* CCUG 49543^T^ used in this study were obtained from the Department of Veterinary and Animal Sciences, Faculty of Health and Medical Sciences, University of Copenhagen. One strain of *S. aureus* ATCC 6538 belonged to the strain collection of the Warsaw University of Life Sciences. *Candida* species was identified based on the positive germ tube test and API Candida (Biomerieux, France). A disk-diffusion method was used to check antimicrobial susceptibility profiles of isolated microorganisms. *Escherichia coli* isolate was tested for susceptibility to amoxicillin with clavulanic acid (AMC; 30 μg), cefpodoxime (CPD; 10 μg), cephalothin (CF; 30 μg), gentamicin (GM; 10 μg), tetracycline (TE; 30 μg), doxycycline (D; 30 μg), sulfamethoxazole with trimethoprim (SXT; 23.75 μg/1.25 μg), florfenicol (FFC; 30 μg), enrofloxacin (ENO; 5 μg), ampicillin (AM; 10 μg), and amikacin (AN; 30 μg), while *S. aureus* isolate was tested for penicillin (P; 10 μg) instead of ampicillin, and it was additionally tested for susceptibility to clindamycin (CC; 2 μg) and erythromycin (E; 15 μg) (Becton Dickinson, USA). The presence of *mecA* gene was checked by PCR method according to Larsen et al. (9). *Candida albicans* isolate was tested for susceptibility to: clotrimazole (CTM; 1 0μg), natamycin (NAT; 10 μg), flucytosine (FY; 1 μg), amphotericin (AMB; 20 μg), ketoconazole (KCA; 10 μg), nystatin (NY; 100 units), and econazole (ECM; 10 μg) (Mast Group, UK). After incubation at 37°C for 24 h, the growth inhibition zones were measured and interpreted in accordance with CLSI guidelines (10, 11).

To evaluate the cumulative data concerning antimicrobial resistance in selected bacteria isolated from pigeons, comprehensive literature search was performed in the PubMed database for studies published from 01.01.2000 to 01.07.2020. The database was searched for the following keywords: bacterial infection, antimicrobial resistance, and pigeon, giving a total of 35 search results. Manual revision and selection of data were based on information in the titles and/or abstracts. Selected publications had to contain extractable data in English on the number of bacterial strains isolated from clinical and/or non-clinical samples from feral and/or domestic pigeons. Moreover, they had to contain data on the resistance profile to the tested antibiotics separately for each tested strain. Considering the fact that among the publications that meet the above criteria, the most numerous were those relating to *E. coli*, 11 publications were selected for the final analysis. Selection of studies and extraction of data were done independently by the authors AG and EK and then compared and reviewed by the third author DCC. The extracted data was collected in a database created for this publication and analyzed for the percentage of strains resistant to particular classes or subclasses of antibiotics. The results obtained in the research on feral pigeons and homing pigeons were also compared.

## Results

In the present study, *S. aureus*, non-hemolytic *E. coli*, and *C. albicans* were isolated from oral cavity of racing pigeon. Disk-diffusion method revealed in *E. coli* isolate intermediate susceptibility to amikacin only. Furthermore, it was resistant to amoxicillin with clavulanic acid, cefpodoxime, cephalothin, gentamicin, tetracycline, doxycycline, sulfamethoxazole with trimethoprim, florfenicol, enrofloxacin, and ampicillin. Whereas, *S. aureus* isolate was resistant to all beta-lactam antibiotics tested and to amikacin, gentamicin, tetracycline, doxycycline, florfenicol, and clindamycin, erythromycin. Intermediate susceptibility was confirmed only to enrofloxacin. The detection of the *mecA* gene in isolated *S. aureus* strain correlated with the antimicrobial resistance phenotype indicating MRSA (methicillin-resistant *S. aureus*). Both bacterial isolates were resistant to at least three antimicrobial classes, thus could be classified as multidrug-resistant pathogens (12).

In mycological examination, *C. albicans* isolate was resistant to clotrimazole, natamycin, flucytosine, and amphotericin. Moreover, it was intermediately susceptible to ketoconazole, nystatin, and econazole.

According to our best knowledge, 10% florfenicol acquired from unknown source was administered orally despite the antibiogram result. The outcome of the disease has remained unknown.

## Discussion

The highlight of this case is the fact that three different pathogenic microorganisms were isolated from an affected racing pigeon, and all of them were multidrug resistant. Although, increasing resistance to antimicrobials in bacteria and fungi is a well-known fact, mistakes in antimicrobial therapy are still common (6, 7). Antibiotics are often administered “blindly,” without previous microbiological examinations, and the drug selection is often random. Antimicrobial therapy must base on the results of antimicrobial susceptibility testing and on the prescription of a veterinarian. In many cases, the antibiotic use is unnecessary because the etiological agent of a disease is not of bacterial origin. Other common problems are wrong dosage of a drug, and too long or too short duration of the treatment. Therapy is often not continued as soon as the clinical symptoms subside. In case of animals taking part in competitive sport, including racing pigeons, before the sporting event, antibiotics are frequently given preventively to treat any possible disease, even if the animal shows no clinical symptoms. Among the domestic pigeon breeders even more irresponsible practices concerning antibiotic usage may occur. Antimicrobial cocktails (preparations consisting of antibiotics from different classes) are purchased from unknown sources and sometimes also shared between breeders. This cocktails can contain not only antimicrobials registered for pigeons or other animals but also antimicrobials registered for humans (13).

The resistance of the *E. coli* isolate to enrofloxacin and doxycycline, as well as the resistance of the *S. aureus* isolate to doxycycline and intermediate susceptibility to enrofloxacin, may be associated with an extensive use of those antimicrobials authorized for treatment of pigeons in Poland. Similar observations were described previously for pigeon pathogens by other research groups (7, 13, 14). However, the resistance to aminoglycosides, macrolides, and phenicols, which are not registered in Poland for use in pigeons, suggests the possible acquisition of resistance determinants from other bacteria, as well as an effect of selective pressure caused by unauthorized previous treatment with antibiotics from these classes. Moreover, we recognized MRSA in the racing pigeon in Poland by PCR with *mecA*-specific primers. Up to date, there is only one report concerning the presence of pigeon methicillin-resistant staphylococci in Poland, but this feature was not genetically confirmed (14). Multidrug-resistant, biofilm-producing *S. aureus* strains were also isolated from pigeons with conjunctivitis in Iran (15). Moreover, in Italy it was shown that pigeons can be colonized by methicillin-resistant *S. aureus* (16).

In this study, we also found multidrug-resistant *E.coli* isolate. It was previously shown that pigeons are reservoir of multidrug-resistant *E. coli*, including ESBL-producing strains (17–19). Cunha et al. (20) found that feral pigeons carried ESBL-positive *E. coli* strains producing the enzymes CTX-M-2 and CTX-M-8 (20).

**Figure 2:**
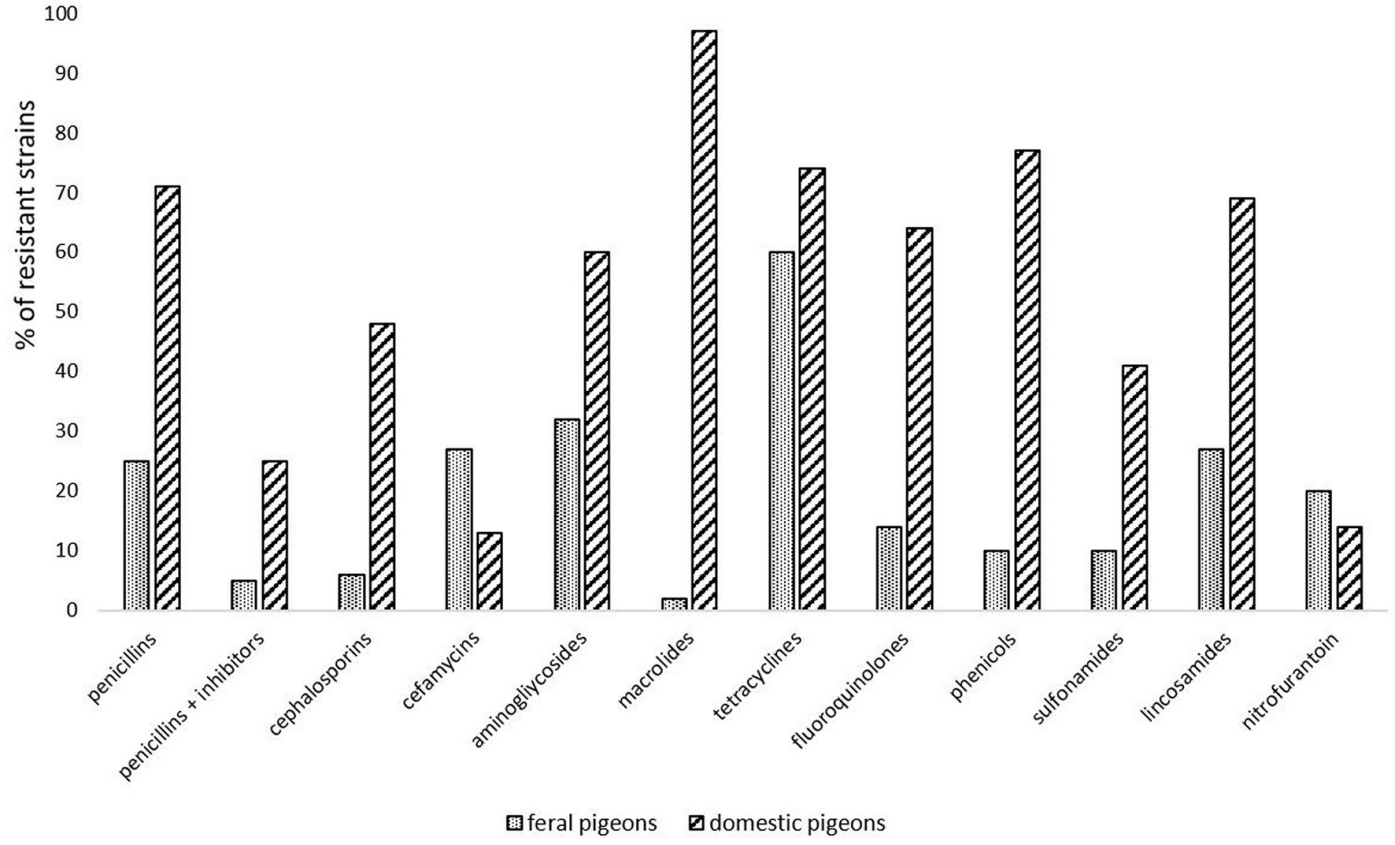
Comparison of antimicrobial resistance in *E. coli* strains isolated from feral and domestic pigeons, according to publications available in the PubMed database (18, 21–30).

Cumulative data based on the analysis of available publications concerning antimicrobial resistance in *E. coli* isolated from pigeons has shown that the majority of them were resistant to tetracyclines. This may be due to the fact, that tetracyclines are registered for birds in many European countries, including Poland. Another class of antimicrobials registered for birds are fluoroquinolones and according to the cumulating data 29% of strains were reported as resistant to them. The highest percentage of strains was resistant to olaquindox; however, data on this antibiotic came only from one study from China (21) (Table 1). Figure 2 compares the differences in resistance to different classes of antibiotics of *E. coli* strains isolated from feral and domestic pigeons. In general, *E. coli* strains obtained from domestic pigeons shown higher rate of resistance to all antimicrobials tested, except nitrofurantoin. However, it is worth noting that most studies on the prevalence of multidrug-resistant zoonotic pathogens concerned feral pigeons, and infectious agents were isolated from faeces of healthy birds. There is only limited data on the isolation of such pathogens from clinical samples, and they are mainly obtained from racing pigeons.

**Table 1:** Cumulative results of antimicrobial resistance in *E. coli* isolated from pigeons, according to publications available in the PubMed database (18, 21–30).

**Antimicrobial or antimicrobial Class**		**% of resistant strains**
Beta-lactams	Penicillins	45
	Cephalosporins	18
	Cefamycins	17
	Penicillins with betalactamase inhibitors	8
Olaquindox		82
Tetracyclines		65
Lincosamides		42
Aminoglycosides		40
Phenicols		32
Fluoroquinolones		29
Macrolides		25
Sulfonamides		17
Nitrofurantoin		17
Tigecycline		3

There is also literature data indicating the presence of multidrug-resistant yeasts in pigeons (31). Multiple studies showed the prevalence of yeasts belonging to the genus *Cryptococcus, Candida, Rhodotorula*, and *Trichosporon* in pigeon droppings (32–36). Moreover, many strains were resistant to the azole antifungal drugs (36). However, as it was described in the case of bacterial isolates, there is only limited data on the isolation of multidrug-resistant yeasts from clinical samples of pigeon origin.

The occurrence of methicillin-resistant staphylococci and other multidrug-resistant microorganisms in pigeons is alarming due to the fact that these pathogens can be transmitted to humans and other animal species. Pigeons may shed such microorganisms in a wide geographical area because the competition flights cover considerable distances (37). Moreover, these birds share the same environment with humans, domestic and wildlife animals, and act as carriers of many emerging pathogens. It is worth to mention that feral pigeons are known to be the source of human pathogens such as toxigenic *E. coli, Salmonella*, and *Enterococcus* (25, 30, 38–42).

The potential risk for public health posed by drastically increasing multidrug resistance of microorganisms isolated from pigeons must be highlighted. However, it must be also emphasized that veterinarians should inform pigeon breeders that multidrug resistance leads to higher morbidity, mortality, and increased treatment costs.

## Data Availability Statement

The original contributions presented in the study are included in the article/supplementary material, further inquiries can be directed to the corresponding author/s.

## Author Contributions

DC-C, AG, and EK contributed to conception and design of the study. BD and KA performed the initial sampling procedure and the collection of isolates. DC-C, EK, and MJB conducted the experiments. DC-C, AG, EK, and MR analyzed the data. DC-C, AG, and EK wrote the draft of the manuscript. MB, MR, and MS critically reviewed sections of the manuscript. All authors contributed to manuscript revision, read, and approved the submitted version.

## Conflict of Interest

The authors declare that the research was conducted in the absence of any commercial or financial relationships that could be construed as a potential conflict of interest.

## References

[1] Sudagidan M Aydin A. Virulence properties of *Staphylococcus delphini* strains isolated from domestic pigeons. Med Wet. (2012) 68:231–36.

[2] Kizerwetter-Swida M Chrobak-Chmiel D Rzewuska M Antosiewicz A Dolka B Ledwoń A. Genetic characterization of coagulase-positive staphylococci isolated from healthy pigeons. Pol J Vet Sci. (2015) 18:627–34. 10.1515/pjvs-2015-0081 26618597

[3] Ozturk Y Celik S Sahin E Acik MN Cetinkaya B. Assessment of farmers' knowledge, attitudes and practices on antibiotics and antimicrobial resistance. Animals (Basel). (2019) 9:653. 10.3390/ani9090653 31487911 PMC6770244

[4] Gibbs EPJ. The evolution of One Health: a decade of progress and challenges for the future. Vet Rec. (2014) 174:85–91. 10.1136/vr.g143 24464377

[5] Destoumieux-Garzón D Mavingui P Boetsch G Boissier J Darriet F Duboz P. The One Health concept: 10 years old and a long road ahead. Front Vet Sci. (2018) 5:14. 10.3389/fvets.2018.00014 29484301 PMC5816263

[6] Ledwoń A Rzewuska M Czopowicz M Kizerwetter-Swida M Chrobak-Chmiel D Szeleszczuk P. Occurrence and antimicrobial susceptibility of *Salmonella* spp. isolated from domestic pigeons *Columba livia* var. domestica in 2007-2017 in Poland. Med Weter. (2019) 75:735–37. 10.21521/mw.6280

[7] Dolka B Czopowicz M Chrobak-Chmiel D Ledwoń A Szeleszczuk P. Prevalence, antibiotic susceptibility and virulence factors of *Enterococcus* species in racing pigeons (*Columba livia* f. domestica). BMC Vet Res. (2020) 16:7. 10.1186/s12917-019-2200-6 31910839 PMC6947970

[8] Sasaki T Tsubakishita S Tanaka Y Sakusabe A Ohtsuka M Hirotaki S. Multiplex-PCR method for species identification of coagulase-positive staphylococci. J Clin Microbiol. (2010) 48:765–69. 10.1128/JCM.01232-09 20053855 PMC2832457

[9] Larsen AR Stegger M Sørum M. *spa* typing directly from a *mecA, spa* and *pvl* multiplex PCR assay-a cost-effective improvement for methicillin-resistant *Staphylococcus aureus* surveillance. Clin Microbiol Infect. (2008) 14:611–4. 10.1111/j.1469-0691.2008.01995.x 18393997

[10] Clinical and Laboratory Standards Institute [CLSI]. Performance Standards for Antimicrobial Disk and Dilution Susceptibility Tests for Bacteria Isolated from Animals. CLSI supplement VET08, 4th ed. Wayne, PA: CLSI (2018).

[11] Clinical and Laboratory Standards Institute [CLSI]. Method for Antifungal Disk Diffusion Susceptibility Testing of Yeasts. CLSI guideline M44, 3rd ed. Wayne, PA: CLSI (2018).

[12] Magiorakos AP Srinivasan A Carey RB Carmeli Y Falagas ME Giske CG. Multidrug-resistant, extensively drug-resistant and pandrug-resistant bacteria: an international expert proposal for interim standard definitions for acquired resistance. Clin Microbiol Infect. (2012) 18:268–81. 10.1111/j.1469-0691.2011.03570.x 21793988

[13] Zigo F Takac L Zigova M Takacova J Vasi M. Occurrence of antibiotic-resistant bacterial strains isolated in carrier pigeons during the race season. J Chem Pharm Sci. (2017) 10:10–13.

[14] Stenzel T Bancerz-Kisiel A Tykałowski B Smiałek M Pestka D Koncicki A. Antimicrobial resistance in bacteria isolated from pigeons in Poland. Pol J Vet Sci. (2014) 17:169–71. 10.2478/pjvs-2014-0023 24724486

[15] Gharajalar SN Shahbazi P. Antimicrobial susceptibility patterns of biofilm forming *Staphylococcus aureus* isolated from pigeon external ocular infections. J Exot Pet Med. (2018) 21:81–4 10.1053/j.jepm.2018.02.006

[16] Losito P Vergara A Muscariello T Ianieri A. Antimicrobial susceptibility of environmental *Staphylococcus aureus* strains isolated from a pigeon slaughterhouse in Italy. Poult Sci. (2005) 84:1802–7. 10.1093/ps/84.11.1802 16463981

[17] Dey RK Khatun M Islam M Hosain M. Prevalence of multidrug resistant *Escherichia coli* in pigeon in Mymensingh, Bangladesh. Microbes Health. (2014) 2:5–7. 10.3329/mh.v2i1.17254

[18] Hasan B Islam K Ahsan M Hossain Z Rashid M Talukder B. Fecal carriage of multi-drug resistant and extended spectrum β-lactamases producing *E. coli* in household pigeons, Bangladesh. Vet Microbiol. (2014) 168:221–24. 10.1016/j.vetmic.2013.09.033 24290770

[19] Cordero J Alonso-Calleja C García-Fernández C Capita R. Microbial load and antibiotic resistance patterns of *Escherichia coli* and *Enterococcus faecalis* isolates from the meat of wild and domestic pigeons. Foods. (2019) 8:536. 10.3390/foods8110536 31683845 PMC6915359

[20] Cunha MPV Oliveira MCV Oliveira MGX Menão MC Knöbl T. CTX-M-producing *Escherichia coli* isolated from urban pigeons (*Columba livia domestica*) in Brazil. J Infect Dev Ctries. (2019) 13:1052–56. 10.3855/jidc.11441 32087078

[21] Yang L Yang L Lü DH Zhang WH Ren SQ Liu YH. Co-prevalance of PMQR and 16S rRNA methylase genes in clinical *Escherichia coli* isolates with high diversity of CTX-M from diseased farmed pigeons. Vet Microbiol. (2015) 178:238–45. 10.1016/j.vetmic.2015.05.009 26013416

[22] Askari Badouei M Zahraei Salehi T Koochakzadeh A Kalantari A Tabatabaei S Molecular characterization, genetic diversity and antibacterial susceptibility of Escherichia coli encoding Shiga toxin 2f in domestic pigeons. Lett Appl Microbiol. (2014) 59:370–76. 10.1111/lam.12288 24863542

[23] Borges CA Maluta RP Beraldo LG Cardozo MV Guastalli EAL Kariyawasam S. Captive and free-living urban pigeons (*Columba livia*) from Brazil as carriers of multidrug-resistant pathogenic *Escherichia coli*. Vet J. (2017) 219:65–67. 10.1016/j.tvjl.2016.12.015 28093116

[24] Ghanbarpour R Daneshdoost S. Identification of shiga toxin and intimin coding genes in *Escherichia coli* isolates from pigeons (*Columba livia*) in relation to phylotypes and antibiotic resistance patterns. Trop Anim Health Prod. (2012) 44:307–12. 10.1007/s11250-011-0021-0 22105907

[25] Kimpe A Decostere A Matrel A Haesebrouck F Devrise LA. Prevalence of antimicrobial resistance among pigeon isolates of *Streptococcus gallolyticus, Escherichia coli* and *Salmonella enterica* serotype Typhimurium. Avian Pathol. (2002) 31:393–97. 10.1080/03079450220141679 12396341

[26] Kumar A Tiwary BK Kachhap S Nanda AK Chakraborty R. An *Escherichia coli* strain, PGB01, isolated from feral pigeon, thermally fit to survive in pigeon, shows high level resistance to trimethoprim. PLoS ONE. (2015) 10:e0119329. 10.1371/journal.pone.0119329 25750990 PMC4353713

[27] Ngaiganam EP Pagnier I Chaalal W Leangapichart T Chabou S Rolain JM. Investigation of urban birds as source of β-lactamase-producing Gram-negative bacteria in Marseille city, France. Acta Vet Scand. (2019) 61:51. 10.1186/s13028-019-0486-9 31672159 PMC6822345

[28] Sacristán C Esperón F Herrera-León S Iglesias I Neves E Nogal V. Virulence genes, antibiotic resistance and integrons in *Escherichia coli* strains isolated from synanthropic birds from Spain. Avian Pathol. (2014) 43:172–75. 10.1080/03079457.2014.897683 24689431

[29] Scullion FT Scullion MG. Multiresistant *Escherichia coli* in racing pigeons. Vet Rec. (2010) 167:880. 10.1136/vr.c6727 21262662

[30] Silva VL Nicoli JR Nascimento TC Diniz CG. Diarrheagenic *Escherichia coli* strains recovered from urban pigeons (*Columba livia*) in Brazil and their antimicrobial susceptibility patterns. Curr Microbiol. (2009) 59:302–8. 10.1007/s00284-009-9434-7 19504156

[31] Lord AT Mohandas K Somanath S Ambu S. Multidrug resistant yeasts in synanthropic wild birds. Ann Clin Microbiol Antimicrob. (2010) 9:11. 10.1186/1476-0711-9-11 20307325 PMC2852373

[32] Teodoro VL Gullo FP Sardi Jde C Torres EM Fusco-Almeida AM Mendes-Giannini MJ. Environmental isolation, biochemical identification, and antifungal drug susceptibility of *Cryptococcus* species. Rev Soc Bras Med Trop. (2013) 46:759–64. 10.1590/0037-8682-0025-2013 24474019

[33] Cafarchia C Camarda A Romito D Campolo M Quaglia NC Tullio D. Occurrence of yeasts in cloacae of migratory birds. Mycopathologia. (2006) 161:229–34. 10.1007/s11046-005-0194-z 16552486

[34] Costa AK Sidrim JJ Cordeiro RA Brilhante RS Monteiro AJ Rocha MF. Urban pigeons (*Columba livia*) as a potential source of pathogenic yeasts: a focus on antifungal susceptibility of *Cryptococcus* strains in Northeast Brazil. Mycopathologia. (2010) 169:207–13. 10.1007/s11046-009-9245-1 19847668

[35] Jang YH Lee SJ Lee JH Chae HS Kim SH Choe NH. Prevalence of yeast-like fungi and evaluation of several virulence factors from feral pigeons in Seoul, Korea. Lett Appl Microbiol. (2011) 52:367–71. 10.1111/j.1472-765X.2011.03009.x 21251028

[36] Magalhães Pinto L de Assis Bezerra Neto F Araújo Paulo de Medeiros M Zuza Alves DL Maranhão Chaves G. *Candida* species isolated from pigeon (*Columbia livia*) droppings may express virulence factors and resistance to azoles. Vet Microbiol. (2019) 235:43–52. 10.1016/j.vetmic.2019.05.022 31282378

[37] Teske L Ryll M Rubbenstroth D Hänel I Hartmann M Kreienbrock L. Epidemiological investigations on the possible risk of distribution of zoonotic bacteria through apparently healthy homing pigeons. Avian Pathol. (2013) 42:397–407. 10.1080/03079457.2013.822468 23930968

[38] Grossmann K Weniger B Baljer G Brenig B Wieler LH. Racing, ornamental and city pigeons carry shiga toxin producing *Escherichia coli* (STEC) with different Shiga toxin subtypes, urging further analysis of their epidemiological role in the spread of STEC. Berl Munch Tierarztl Wochenschr. (2005) 118:456–63. 16318269

[39] Sonntag AK Zenner E Karch H Bielaszewska M. Pigeons as a possible reservoir of Shiga toxin 2f-producing *Escherichia coli* pathogenic to humans. Berl Munch Tierarztl Wochenschr. (2005) 118:464–70. 16318270

[40] Cizek A Literak I Hejlicek K Treml F Smola J. Salmonella contamination of the environment and its incidence in wild birds. J Vet Med B. (1994) 41:320–27. 10.1111/j.1439-0450.1994.tb00234.x 7839754

[41] Oliveira MCV Camargo BQ Cunha MPV Saidenberg AB Teixeira RHF Matajira CEC. Free-ranging synanthropic birds (*Ardea alba* and *Columbia livia* domestica) as carriers of *Salmonella spp.* and diarrheagenic *E. coli* in the vicinity of an urban zoo. Vector Borne Zoonotic Dis. (2018) 18:65–9. 10.1089/vbz.2017.2174 29261025

[42] Capita R Cordero J Molina-González D Igrejas G Poeta P Alonso-Calleja C. Phylogenetic diversity, antimicrobial susceptibility and virulence characteristics of *Escherichia coli* isolates from pigeon meat. Antibiotics (Basel). (2019) 8:259. 10.3390/antibiotics8040259 31835475 PMC6963593

